# Corrosion Behavior of AISI 1018 Carbon Steel in Localized Repairs of Mortars with Alkaline Cements and Engineered Cementitious Composites

**DOI:** 10.3390/ma13153327

**Published:** 2020-07-27

**Authors:** Erick Maldonado-Bandala, Noema Higueredo-Moctezuma, Demetrio Nieves-Mendoza, Citlalli Gaona-Tiburcio, Patricia Zambrano-Robledo, Héctor Hernández-Martínez, Facundo Almeraya-Calderón

**Affiliations:** 1 Facultad de Ingeniería Civil, Universidad Veracruzana, Xalapa, 91000 Veracruz, Mexico; eemalban@gmail.com (E.M.-B.); dnieves@uv.mx (D.N.-M.); hehernandez@uv.mx (H.H.-M.); 2 Facultad de Ingeniería Mecánica Eléctrica, Universidad Veracruzana, Xalapa, 91000 Veracruz, Mexico; noema_higueredo@hotmail.com; 3 Universidad Autonoma de Nuevo Leon, FIME—Centro de Investigación e Innovación en ingeniería Aeronáutica (CIIIA), Av. Universidad s/n. Ciudad Universitaria, San Nicolás de los Garza, Nuevo León 66455, Mexico; citlalli.gaona@gmail.com (C.G.-T.); patricia.zambranor@uanl.edu.mx (P.Z.-R.)

**Keywords:** repair mortar, corrosion, engineered cementitious composites, alkali-activated, supplementary cementitious materials, electrochemical techniques

## Abstract

The selection of materials for repairs of reinforced concrete structures is a serious concern. They are chosen for the mechanical capacity that the repair mortar achieves. However, several important characteristics have been left aside, such as the adhesion of the repair mortar with the concrete substrate, the electrical resistivity and—hugely important—the protection against corrosion that the repair material can provide to the reinforcing steel. The aim of this work was to study the corrosion behavior of AISI 1018 carbon steel (CS) in mortars manufactured with alkaline cements, engineered cementitious composites (ECC), and supplementary cementitious materials (SCM). Two types of ordinary Portland cement (OPC) 30R and 40R were used. The constituent materials for the mortars with ECC mixture mortars they use OPC 40R, class F fly ash (FA), silica fume (SF) and polypropylene (PP) fibers. The sodium hydroxide (NaOH) and sodium silicate (Na_2_SiO_3_) were used as activating agents in alkali activated cements. The reinforced specimens were immersed in two different electrolytes, exposed to a 3.5 wt % of NaCl and Na_2_SO_4_ solutions, for 12 months and their electrochemical behavior was studied by half-cell potential (E_corr_) and linear polarization resistance (LPR) according to ASTM C876-15 and ASTM G59-97, respectively. The results obtained indicated that, the mortar they have the best performance and durability, is the conventional MCXF mortar, with OPC 30R and addition of 1% polypropylene PP fiber improves the behavior against the attack of chlorides and sulfates.

## 1. Introduction

Portland cement production is a high energy demand process [1,2] that emits 900 kg CO_2_ into the atmosphere per tonne of cement [3] which contributed to global warming and climate change. Therefore, in looking for ways to reduce CO_2_ emissions associated with cement production [4,5] by the use of supplementary cementitious materials (SCM) to replace part of Portland cement [6].

The corrosion of reinforcing steel in concrete can be subject to multiple causes of potential damage and deterioration of the infrastructure [7,8], which can be physical, mechanical, biological, or chemical. However, the main problem for which a reinforced concrete structure requires repair is the corrosion of steel, negatively influencing the durability of the constructions, putting their functionality and safety at risk [9], inducing cracking, concrete release, and mechanical weakening. Once reinforcing bars are exposed to the environment, corrosion occurs at higher velocity that leads to the loss of the reinforcement steel area [10,11,12,13]. The durability of reinforced mortar is demerited with the corrosion of the reinforcement, with determining factors such as the structure and pore distribution and mechanical strength [14]. Different solutions to retard or reduce the corrosion process and mitigate emissions produced from the cement industry have been investigated. The new alkali-activated materials have replaced the OPC with different types of ashes with the purpose of providing durability to mortars, with satisfactory results. The replacement of OPC by ashes: fly ash (FA), slags furnace ash (SFA), and metakaolin (MK) among others [15,16]. Furthermore, in the last 20 years, sugar cane bagasse ash (SCBA), and rice husks ash (RHA), have been studied in order to provide a more sustainable and equally performing solution for reinforced structures [17,18,19,20], as it decreases the effects of corrosion of the reinforcement produced in harsh environments and improves mechanical, structural, physical, chemical, and electrochemical properties [21,22].

After having detected the damage by corrosion in a structure, it is necessary to take actions to develop a repair and extend its useful life. Some of the most studied materials to perform these repairs are repair mortars commonly made with ordinary Portland cement (OPC) mortars, engineered cementitious composites (ECC), with pozzolanic additions such as fly ash (FA) or silica fume (SF). Several tests have been carried out to evaluate only the mechanical behavior [23,24,25], and under static load conditions, greater resistance to cutting has been observed [26,27,28] which in turn they have shown greater tolerance to cyclic loads [29,30]. In addition to this, the cement matrix has incorporated polyvinyl alcohol (PVA) or polyethylene (PE) fibers, demonstrating that these compensate for the efforts of a corroded steel bar to repair reinforced concrete beams under bending stresses [31]. On the other hand, mortars with alkali-activated metakaolin (MK) have been developed [32,33,34]. However, studies that have been conducted on this topic are scarce based solely on their thermal properties [35], chemical [36], and mechanical [20,37]. Also, its properties have also been studied as protection against fires in structures [38,39].

It is common to find bad repairs made with conventional mortars of poor quality, using high water/cement ratios (w/c) and dissimilar materials to the structure to be repaired, which only aggravate the problem in the future by accelerating the corrosion in the bar reinforcing steel. It is also important to note that there have been no studies on the corrosion protection provided by mortars made with ECC added with FA and SF, also there are no studies on mortars with alkaline activated metakaolin (MK) and mortars with metakaolin/sugar cane bagasse ash (MK/SCBA) [8,16,19,20].

The aim of this work was to study the corrosion behavior of carbon steel in mortars with alkaline cements and engineered cementitious composites. They were compared three conventional mortars, with two repair mortars made with ECC where the cement is replaced by 20% by FA and SF; and two more with total replacement of OPC by MK and MK/SCBA. The reinforced specimens were immersed in two different electrolytes, exposed at 3.5 wt % of NaCl and Na_2_SO_4_ solutions, for 12 months. The electrochemical corrosion was studied by half-cell potential (E_corr_) and linear polarization resistance (LPR) according to ASTM C-876-15 and ASTM G59-97, respectively.

## 2. Materials and Methods

### 2.1. Materials

Ordinary Portland cement (OPC) of two classifications was used; OPC 30R was used according to NMX-C-414-ONNCCE [40] of 30 MPa and OPC 40R [40] of 40 MPa at 28 days. The pozzolanic additions were made with Class F Fly Ash (FA) type according to ASTM C618-94a [41], obtained from a thermoelectric plant in Coahuila, Mexico. It was used metakaolin (MK) which was obtained by calcination of mineral kaolinite at 750 °C for 4 h. Finally, the sugarcane bagasse ash was obtained from the sugar industry in the Veracruz, Mexico. The chemical composition of the materials is shown in Table 1.

Were designed six repair mortars and one for control.

- Two mortar with OPC 30R (MCX and MCXF) which was manufactured in conditions similar to the control but varying the ratio w/c and adding polypropylene (PP) fibers.
- Two mortars with OPC-40R adding polypropylene fibers more fly ash (MECC-FA) and other with silica fume (MECC-SF).
- Two mortars were manufactured with 100% metakaolin (MK), and another in equal proportions of MK/SBCA. The sodium hydroxide (NaOH) and sodium silicate (Na_2_SiO_3_) were used as activating agents in alkali activated cements. In Table 2, the formulation of the alkali activated cements is observed. The detailed mix proportion of mortar is also shown in Table 3.

The PP fiber used in the present study has an average diameter of 39 µm, and a length of 12 mm nominal. The tensile strength and maximum elongation of the fiber are 6120 MPa and 6.0% respectively. The fibers are coated with 1.2% (by weight) proprietary hydrophobic agent to modify the interaction between the fiber and cement matrix for better performance.

The specimens were placed in the lower part the concrete substrate and in the upper part the repair mortar.

The compressive strength of mortars was measured according to ASTM C39 [42], by 30 t class universal testing machine (Controls Mod. Pilot. Milan, Italy), and the average value was used by measuring the compressive strength in a triplicate set of specimens at 7, 14, 60 and 360 days.

### 2.2. Test Methods

The corrosion evaluation of AISI 1018 carbon steel in structural repair mortars was developed by comparing the performance against corrosion of a control mortar (MC) which was manufactured by means of conventional masonry techniques against six mortars of structural repair.

#### 2.2.1. Electrochemical Techniques

**Figure 1:**
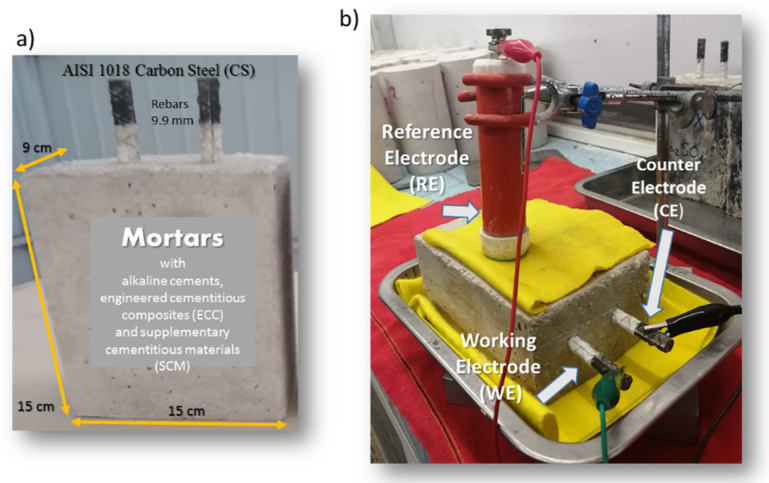
Experimental arrangement. (**a**) Specimens dimensions, (**b**) conventional three-electrode corrosion cell.

Prismatic mortar specimens with dimensions of 9 × 15 × 15 cm (Figure 1a) were manufactured with two rebars embedded in the mortars. The AISI 1018 carbon steel (CS) was used as bars and had a length of 15 cm and 9.5 mm diameter. A conventional three-electrode cell configuration (Figure 1b) was used for the electrochemical studies, which consisted of a two nominally identical electrodes (CS bars) as working electrode (WE) and counter electrode (CE), a copper–copper sulfate (Cu/CuSO_4_) electrode, respectively. Electrochemical measurements were carried out using a Gill-AC potentiostat/galvanostat/ZRA from ACM Instruments (Manchester, UK). Corrosion experiments were performed by immersion in 3.5 wt % of NaCl and Na_2_SO_4_ solutions, for 12 months, at room temperature. The electrochemical tests were performance in triplicate.

The half-cell corrosion potential (E_corr_) was used to assess the corrosion condition of reinforced concrete specimens according to ASTM C876-15 Standard [43] and considering one more range, according to the literature [44]. E_corr_ establishes the criteria or ranges that relate the E_corr_ values with the corrosion risk for embedded steel specimens made mortars with alkaline cements and engineered cementitious composites, see Table 4 [43,44].

Linear polarization resistance (LPR) were recorded at a sweep rate of 10 mV/min at, a potential scan range was applied between −20 to +20 mV vs. (Cu/CuSO_4_), according to ASTM G59-99 Standard and Stern–Geary [45,46]. The *i*_corr_ and the corrosion rate (*v*_corr_) were estimated from the LPR technique using the Stern and Geary equation [46]

(1) $$i_{corr}=\frac{\beta }{Rp}$$

where *Rp* is expressed in Ω.cm^2^ and B in V is a constant resulting from a combination of the anodic and cathodic Tafel slopes: B is a constant with recommended value of 0.026 V for active and 0.052 V for the passive corrosion of steel in concrete [47,48]. Corrosion levels were defined according to the Durar network specifications [49].

To determine *v*_corr_ of carbon steels embedded in the specimens made mortars with alkaline cements and engineered cementitious composites, the *i*corr values were used. The criteria used to analyze the *i*corr results are based on the state of corrosion of steel in concrete reported in the literature [50], as shown in Table 5.

#### 2.2.2. Electrical Resistivity and Adherence

The resistivity tests were made according to UNE 83988-2 standard [51] (four-point method: Wenner’s method) at 14, 28, 60, and 360 days. This method consists of using an equipment that has four electrodes spaced by equal distance (5 cm). Being positioned on the surface of the concrete, an electrical charge is applied between the two external electrodes, and the two internal electrodes measure the resulting difference of potential. Three specimens for each mixture were used and three resistivity measurements were made in each specimen, spaced by 120° angles; for each condition, a total of nine measurements were taken.

The values of electrical resistivity needed to be corrected according to the size of the specimens. This factor is 0.377. Thus, the choice of the shape of samples (10 cm diameter and 20 cm height) occurred due to previous knowledge of the form factor necessary for measuring electrical resistivity. Furthermore, the adhesion with slant shear test was evaluated [52]. Cylinders of 10 cm in diameter and 20 cm in length were used, made of two identical halves joined to 30° and tested under axial compression, the lower half was the concrete to be repaired (concrete substrate 30 MPa) and the upper half of the repair mortars analyzed (Table 6).

## 3. Results and Discussion

### 3.1. Compressive Strength

**Figure 2:**
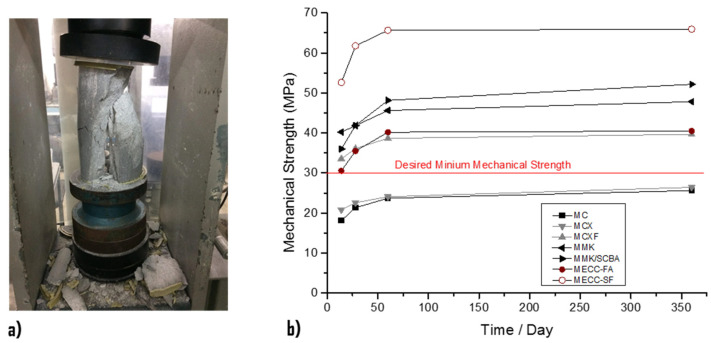
Compressive strength of mortars. (**a**) Test according to ASTM C39 [42]. (**b**) Measuring at ages 7, 14, 60, and 360 days.

The results obtained from simple compressive strength (Figure 2) of the analyzed repair mortars are observed. At an early age, the conventional mortar MCX has a better compressive strength than the MC mortar, since this mortar had an w/c = 0.45 ratio and the mortar MC has a w/c = 0.78 ratio. At the age of 90 days, the compressive strength of both mortars does not have a significant difference because the MCX mortar had a resistance of 25.63 MPa and the MC a resistance of 26.42 MPa, both of which do not reach the desired minimum strength of the concrete substrate.

In the mortar with MCXF fibers, it is observed that the addition of 1% fiber with respect to the weight of cement significantly increased the compressive strength by 39.65 MPa with respect to the MC and MCX mortars. That means that the addition of 1% PP fiber recorded an increase in compressive strength of 13.23 MPa at the age of 90 days. The results indicated that the MECC-FA mortar presents lower compressive strengths than the mortar with MCXF fibers at early ages. However, at the age of 90 days, it registered an increase in its resistance of 40.51 MPa; that is, 0.86 MPa in mortars with MCXF PP fiber.

High-tenacity polypropylene (PP) fiber was studied as an alternative to polyvinyl alcohol (PVA) fiber because of its lower cost and energy intensity [53,54]. PP ECC was found to have ductility (3% strain capacity) comparable to that of PVA ECC [55]. ECC with hybrid PVA and PP fibers showed improved ductility with lower strength [56].

MECC-SF structural repair mortar is the mortar that achieved the highest compressive strength resulting in a 90-day compressive strength of 65.92 MPa. In addition, extremely high compression resistances were observed from early ages, since at 14 days it developed compression resistances of 52.60 MPa. 

The mortars manufactured with alkaline cements MMK and MMK/SCBA obtained higher compression strengths than the MC, MCX, MCXF, and MECC mortars, although they were inferior to the MECC-SF structural repair mortar. The resistance of the mortars with alkaline cements at 90 days was approximately 50 MPa.

### 3.2. Half-Cell Potential and Current Density 

**Figure 3:**
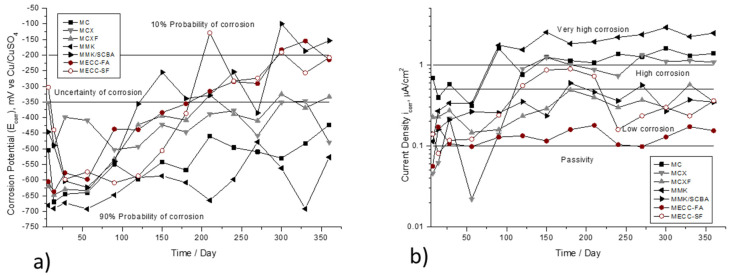
Corrosion behavior of AISI 1018 CS in mortars exposed in 3.5 wt % NaCl solution. (**a**) Half-cell potential (E_corr_). (**b**) Current density (i_corr_).

Figure 3 shows the variation of E_corr_ and i_corr_, as a function of the immersion time in 3.5 wt %. NaCl solution. In Figure 3a that the elements repaired with conventional mortar MC with relation to w/c 0.78 have potential values less than −400 mV, indicating 90% probability of corrosion, the specimens with mortar MCX and MCXF do not present significant change because both have values of high probability of corrosion but at 200 days, show potentials close to −350 mV. It is important to highlight the values of the elements repaired with MECC-FA, MCCE-SF, and MMK/SCBA mortars since at 150 days, the steel rod starts to passivate and has values higher than −350 mV, and at approximately 300 days, has a 10% probability of corrosion. Otherwise, it happened with the elements repaired with mortar based on activated cement MMK that presented the values of E_corr_ more electronegative.

Figure 3b shows the current density values. The behavior of the repaired specimens with the mortars MCXF, MECC-FA, MCCE-SF, and MMK/SCBA, from 25 days to 365 days, have values between 0.1 and 0.5 μA/cm^2^, placing themselves in the ranges of low corrosion. In this respect, Andrade and González [57] describe that it is precisely in this range of values of i_corr_ that the depassivation of the reinforcement is initiated. The other specimens presented very high values of current density in high and very high corrosion ranges. This is possibly due to the high alkali demand and the high specific surface area of MK, which leads to a high demand for water and, consequently, a high contraction and cracking [58] and in the presence of water, allows the entry of Cl^−^ ions more easily.

**Figure 4:**
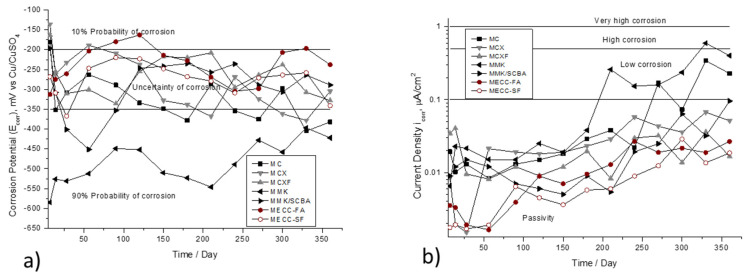
Corrosion behavior of AISI 1018 CS in mortars exposed in 3.5 wt % Na_2_SO_4_ solution. (**a**) Half-cell potential (E_corr_). (**b**) Current density (i_corr_).

Figure 4 shown the E_corr_ and i_corr_ variances, as a function of the immersion time in 3.5 wt %. Na_2_SO_4_ solution. The Figure 4a observed that the specimens repaired with mortar based on alkaline activated cement with metakaolin MMK presents the most electronegative values, between −600mV at the beginning of the monitoring up to −400 mV at 365 days, placing in a region of 90 % probability of corrosion. In the corrosion kinetics presented in Figure 4b, where this same specimen presents the highest corrosion rate (values greater than 0.5. μA/cm^2^), after 200 days it is depassivated and increases the corrosion rate.

The discuss the i_corr_ data is by using the accumulated corrosion graphs (μA/cm^2^ × month), which results from calculating the area under the i_corr_ curve over time. This form of representation has the advantage that the accumulated corrosion, calculated by integrating the rate up to the appropriate age, always grows with time and allows a better comparison of the effects. This tool has been previously reported in this field [13,59,60,61,62]. The reported information discussed by these authors contains not only the use of cumulative corrosion, but also their interpretation in both natural and simulated tests.

**Figure 5:**
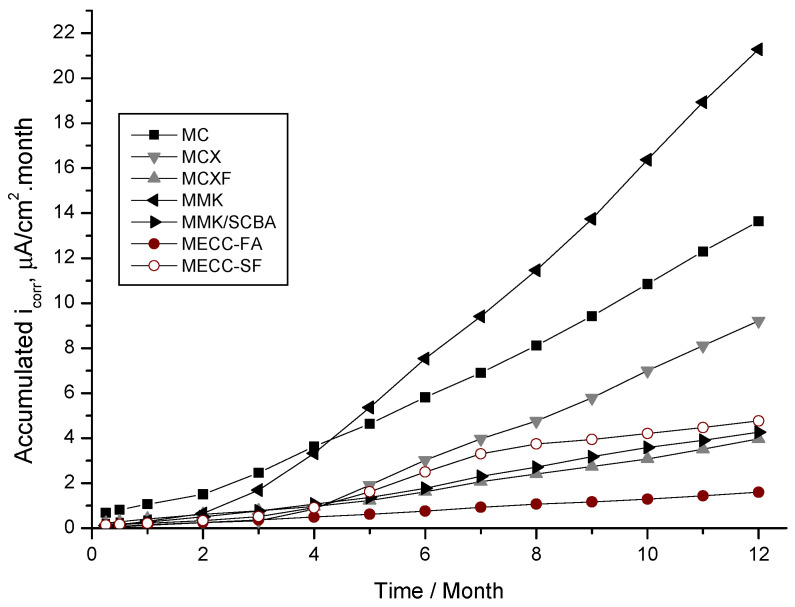
Evolution of the accumulated corrosion current density of AISI 1018 CS in mortars, exposed in 3.5 wt % NaCl solution.

Figure 5 shows the evolution of accumulated corrosion current density of rebar’s exposed in a 3.5 wt % NaCl solution, where it is more evident that the specimens repaired with a conventional mortar MC have a very high corrosion rate. However, those manufactured with MMK present an inflection point greater than 4 months (approximately 120 days), demonstrating very low protection against corrosion of the reinforcement steel. It was observed that, at 3 months (90 days), there is a change of slope in the graph for this two mortars MC and MMK, which is attributed to the depassivation period, which can be related to what was observed in this same period in Figure 4b.

The behavior of the accumulated corrosion current density in the AISI 1018 CS of the specimens repaired with MECC-FA mortar, show a slope tending to the horizontal, which is considered as negligible corrosion. The mortars MCXF, MMK/SCBA, and MECC-FA showed very similar slopes without deflections or sudden changes, indicating that the accumulated corrosion during the test time was very low. Therefore, these mortars can be considered as good protectors against the corrosion of reinforcing steel in chlorides and sulfates solutions.

**Figure 6:**
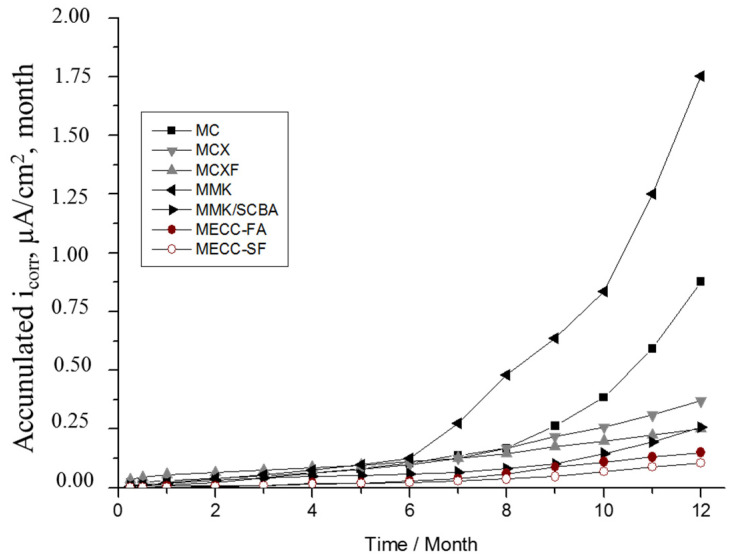
Evolution of the accumulated corrosion current density of AISI 1018 CS in mortars, exposed in 3.5 wt % Na_2_SO_4_ solution.

Figure 6 shows the evolution of accumulated corrosion current density of rebar’s of exposed in 3.5 wt % Na_2_SO_4_ solution. The mortar that offers less protection against corrosion is the MMK due to the steep slopes that showed accumulated corrosion, highlighting a change of values in month 6. By making the comparison with the graph of the Figure 4b, this change is evident the moment in which it crosses the range of passivity and pass to low corrosion and presents values greater than 0.1 μA/cm^2^. The MCXF, MMK/SCBA, and MECC-FA mortars are the ones that present the best behaviors against the corrosion of reinforcing steel.

With the results obtained in this research, diagrams can be constructed that relate the electrical, mechanical, and corrosion properties.

Electrical resistivity was evaluated as a parameter of durability of the repair mortars, so that this technique can be used to establish the relationship with properties, such as: degree of hydration of the cement [63], resistance to compression [64,65,66,67,68,69,70], resistance to permeability to Cl^−^ ion [71], setting time [72], and probability of corrosion [73]. The electrical resistivity can be indirectly related to the porosity of the mortar or concrete and, therefore, may be linked to the susceptibility of the entrance of aggressive agents.

The resistance to the bond between concrete substrate and repair mortar has a great influence because it can represent a very weak link and can be considered as the area where aggressive agents enter to trigger corrosion damage [52]. That perspective implies that if there is good adherence between old concrete and repair mortar, the failure presented in the test will be monolithic, that is, as an entire piece. If the failure is due to the interface or union between substrate concrete and repair mortar, the failure is considered by adhesion [74], which is critical because it generates an area of aeration that allows the entry of chloride ions.

**Figure 7:**
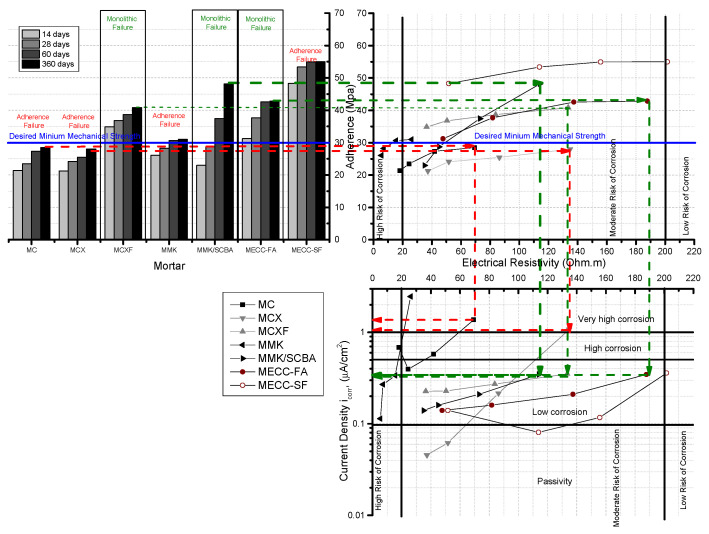
Relation between electrical resistivity, adherence, and corrosion current density of AISI 1018 CS in mortars exposed in 3.5 wt % NaCl solution.

In the Figure 7, this diagram only presents mortars exposed in 3.5 wt % NaCl solution, this diagram relationship the adhesion tests of the mortars studied with the proposed concrete substrate, by using the slant shear method vs. electric resistivity vs. current density, obtained by the LPR method. It can be seen that the green lines correspond to the specimens repaired with MCXF, MMK/SCBA, and MEC-FA mortars, which showed an adequate adherence with the old concrete (substrate concrete) with a monolithic failure.

Likewise, the values of electrical resistivity at 365 days were placed in ranges of moderate probability of corrosion according to the Durar Network: Red Durar [28]. Furthermore, it is also well known that these three repair mortars have corrosion rates in the range of 0.1 to 0.5 μA/cm^2^. The specimens MC, MCX, and MMK, which present 365 days after testing a failure of adhesion, that is to say for the union between mortar-concrete, and resistances much lower than those estimated of 30 MPa, are located in values of resistivity between 40 and 140 Ω m, but with very high corrosion rates. This diagram is quite useful for selecting the repair materials to be used by applying not only mechanical criteria, but also electrical and electrochemical.

The MMK/SCBA repair mortar presented a good result and can be attributed to the presence of a higher silica content (Si:Al ratio) than the MMK mortar. A higher silica content is generally beneficial, experimental evidence suggests that it can lead to smaller pore formation that may prevent the entry of aggressive agents in the long term [75].

The MECC-FA and MECC-SF mortars presented results. The results may be caused by secondary reactions of hydration of Portland cement and the high percentage of SiO_2_ in the SCBA. Therefore, it results in a denser cement matrix with fewer pores that prevents the rapid entry of aggressive agents (NaCl and Na_2_SO_4_) and the same compact structure of hydrated calcium silicates (CSH) provides a greater advantage in terms of mechanical properties and corrosion.

The CSH is responsible for the hardness and compactness of the concrete, the SiO_2_ of the SCBA and the pozzolan reacts as follows: First reaction of hydration (first production of CSH):

2(3CaO·SiO_2_) + 6H_2_O → 3CaO·2SiO_2_·3H_2_O + 3Ca(OH)_2_. (2)

Second reaction of hydration after 14 days of conventional curing (second production CSH):

3Ca(OH)_2_ + 2SiO_2_ + H_2_O → 3CaO·2SiO_2_·3H_2_O. (3)

The performance of the conventional MCXF mortar, which was manufactured with ordinary Portland cement of 30 MPa, and ratio w/c of 0.78, presented good protection against corrosion due to the incorporation of polypropylene fibers. According to consulted literature, they diminish the propagation of micro cracks in the hardening stage of cement [75].

The engineered cementitious composites with supplementary cementitious materials, such as fly ash (FA) and silica fume, have been extensively studied, their use can reduce the carbon and energy footprints [76,77,78].

## 4. Conclusions

- The use of corrosion, electrical resistivity and adhesion tests allowed observing the behavior AISI 1018 carbon steel (CS) in mortars manufactured with alkaline cements and engineered cementitious composites (ECC) adding supplementary cementitious materials (SCM)
- The mortar which has the best performance and durability is the conventional MCXF mortar, with OPC 30R and addition of 1% polypropylene PP fiber improves the behavior against the attack of chlorides and sulfates. In addition, it improved its resistance to compression giving results comparable to the mortar MECC-FA. The failures presented in adhesion were monolithic.
- The mortars with alkali-activated cement metakaolin MK have high compressive strengths. However, the values of electrical resistivity and corrosion were poor, placing it in an area of high risk of corrosion.
- The mortars MECC-FA and MMK/SCBA have excellent durability properties. However, the use of conventional mortars such as MCXF—which develops good durability properties—can be implemented.
- The proposed diagram in Figure 7 can be used as an efficient tool to relate the adhesion, electrical resistivity, and corrosion. It can also help in the selection of mortar or materials that will be used for the repair of reinforced concrete structures.

## Figures and Tables

**Table 1:** Chemical composition of the materials (wt %).

Materials	SiO_2_	Al_2_O_3_	Fe_2_O_3_	CaO	MgO	Na_2_O	K_2_O	SO_3_	MnO	TiO_2_
Ordinary Portland cement (OPC)	18.47	4.13	3.80	65.31	1.42	0.46	1.13	4.64	0.19	0.29
Sugarcane bagasse ash (SCBA)	66.91	6.50	3.88	4.33	2.38	1.06	10.18	0.059	0.19	0.58
Metakaolin (MK)	73.24	22.67	0.15	0.04	-	0.06	0.46	0.14	-	0.275
Fly ash (FA)	57.3	28.14	5.21	3.26	0.56	0.51	1.52	0.32	-	1.21
Silica fume (SF)	95	0.71	0.12	0.43	1.13	-	-	0.18	-	-

**Table 2:** Formulation of activated cements.

Alkali Activated Cement	SCBA (%)	MK (%)	NaOH (%)	Silicate Modulus (SM)	Blaine Surface Area (m^2^/kg)	Initial Curing Temperature (°C)
MK	0	100	14	1	600	600
MK/SCBA	50	50	16	1.5	600	300

**Table 3:** Mortar mixture proportion (%).

Mortars	Description of Mortar	OPC-30R	OPC-40R	Water	Silica Sand	Additive	Fiber (wt %)	MK	SCBA	FA	SF
MC	Portland cement w/c = 0.78	1.0	-	0.78	2.7	-	-	-	-	-	-
MCX	Portland cement w/c = 0.45	1.0	-	0.45	2.7	0.02	-	-	-	-	-
MCXF	Portland cement w/c = 0.45 + fiber 1.0 wt %	1.0	-	0.45	2.7	0.02	1.0	-	-	-	-
MMK	Alkali activated MK cement	-	-	0.45	2.7	0.14	-	1.0	-	-	-
MMK/SCBA	Alkali activated MK/SCBA cement	-	-	4.45	2.7	0.14	-	0.5	0.5	-	-
MECC-FA	Engineered cementitious composites (FA)	-	0.8	0.18	-	0.02	2.2	-	-	0.20	-
MECC-SF	Engineered cementitious composites (SF)	-	0.8	0.15	-	0.02	2.2	-	-	-	0.20

**Table 4:** Measured half-cell corrosion potential (E_corr_) versus a Cu/CuSO4 in reinforcement concrete.

E_corr_ vs. Cu/CuSO_4_ (mV)	Corrosion Activity
E_corr_ > −200	10% probability of corrosion
−200 > E_corr_ > −350	Uncertainty of corrosion
E_corr_ < −350	High corrosion probability of 90%

**Table 5:** Ranges of corrosion current density (*i*_corr_), and the corrosion rate (*v*_corr_) related to corrosion level [49].

*i*_corr_ (µA/cm^2^)	*v*_corr_ (mm/d)	Corrosion Level
*i*_corr_ ≤ 0.1	*v*_corr_ ≤ 0.001	Negligible (passivity)
0.1 < *i*_corr_ < 0.5	0.001 < *v*_corr_ < 0.005	Low corrosion
0.5 < *i*_corr_ < 1	0.005 < *v*_corr_ < 0.010	Moderate corrosion
*i*_corr_ > 1	*v*_corr_ > 0.010	High corrosion

**Table 6:** Variables tested in this study.

**Electrochemical Characterization**				
**Variable**	**Level**	**Description**	**Repetitions**	**Response**
Type of repair mortar	7	MC MCX MCXF MMK MMK/SCBA MECC-FA MECC-SF	2	Corrosion Potential (E_corr_) Corrosion Density (i_corr_)
Time elapsed since production	15	Distributed in 360 days	-	-
Exposure medium	3	Potable water 3.5 wt % NaCl solution at 23 °C 3.5 wt % Na_2_SO_4_ solution at 23 °C	-	-
**Mechanical Properties**				
**Variable**	**Level**	**Description**	**Repetitions**	**Response**
Type of mortar	7	MC MCX MCXF MMK MMK/SCBA MECC-FA MECC-SF	5	Compressive Strength (f’c) Electrical Resistivity (Ω⋅m)
Time elapsed since production	-	7, 14, 28, 60, and 360 days	-	-
Exposure medium	1	Curing room at 90% RH and 23 °C	-	-
